# Mental health-related quality of life in mothers of children with surgically repaired congenital heart disease: a 13-year longitudinal study

**DOI:** 10.1007/s11136-023-03440-y

**Published:** 2023-05-30

**Authors:** Melanie Ehrler, Corina Wettach, Ingrid Beck, Emanuela R. Valsangiacomo Buechel, Beatrice Latal, Markus A. Landolt

**Affiliations:** 1 Child Development Center, University Children’s Hospital Zurich, Zurich, Switzerland; 2 Children’s Research Center, University Children’s Hospital Zurich, Zurich, Switzerland; 3 URPP Adaptive Brain Circuits in Development and Learning, University of Zurich, Zurich, Switzerland; 4 Department of Pediatrics, Children’s Hospital of Eastern Switzerland, St. Gallen, Switzerland; 5 Division of Cardiology, Pediatric Heart Center, University Children’s Hospital Zurich, Zurich, Switzerland; 6 Department of Psychosomatics and Psychiatry, University Children’s Hospital Zurich, Zurich, Switzerland; 7 Division of Child and Adolescent Psychology, Department of Psychology, University of Zurich, Zurich, Switzerland

**Keywords:** Quality of life, Well-being, Mothers, Parents, Congenital heart disease, Longitudinal study

## Abstract

**Aims:**

Having a child with congenital heart disease (CHD) can affect parental health-related quality of life (HR-QoL). We investigated the long-term trajectories of mental HRQoL (m-HRQoL) in mothers of children with CHD and examined risk factors for persistent low m-HRQoL.

**Methods:**

One hundred twenty-five mothers of children with CHD completed a standardized questionnaire on m-HRQoL (mental subscale SF-12) after the children’s first open-heart surgery and subsequently when the children were 1, 4, 6, 10, and 13 years old. A z-score for m-HRQoL was calculated with national norms. Latent class growth analysis (LCGA) was used to identify subgroups of mothers with regards to their m-HRQoL trajectories over time. Regression analysis investigated predictors for chronically low m-HRQoL.

**Results:**

Compared to norms, mothers of children with CHD had significantly lower m-HRQoL immediately after open-heart surgery (*β* = −0.30 (*CI-95:* −0.44, −0.15)). Subsequently, m-HRQoL increased to a normal level (m-HRQoL compared to the norm from 1 to 13 years: *β* ranges between 0.05 and 0.27). LCGA revealed two distinct groups of m-HRQoL trajectories: A group with normal m-HRQoL (75% of mothers, *means* z-scores range between − 0.76 and 0.62) and a group with chronically low m-HRQoL (25% of mothers, *mean z-scores* range between −1.32 and −0.10). Chronically, low m-HRQoL was associated with poorer social support (*OR* = 3.39 (*CI-95:* 1.40, 8.49), *p* = 0.008) but not with parental education, migration background, number of open-heart surgeries, diagnosis of a univentricular CHD, or low IQ.

**Conclusion:**

A quarter of mothers of children with CHD have chronically low m-HRQoL throughout their child’s development, especially those mothers with poor social support. Further studies of family-oriented approaches are needed to identify and support these mothers and reinforce parental well-being.

**Supplementary Information:**

The online version contains supplementary material available at 10.1007/s11136-023-03440-y.

## Plain English summary

Having a child with congenital heart disease (CHD) can substantially affect their parents’ mental health-related quality of life (m-HRQoL). Mothers of children with CHD often report low m-HRQoL when their children undergo surgery. One in four mothers continue to have low m-HRQoL throughout their children’s development into adolescence. Especially mothers with sparse social support have low m-HRQoL in the long term. Family-based interventions targeting families with low psychosocial support may aid and reinforce parental well-being in the long term.

## Introduction

Having a child with congenital heart disease (CHD) puts parents at risk for impaired mental health and reduced quality of life (QoL). When a child is born with CHD, the parents experience shock, disbelief, despair, and grief [1]. Shortly after birth, many infants with CHD undergo medical and surgical interventions, intensive care unit (ICU) treatment, and overall prolonged hospitalization [2]. Reasonably, this traumatic experience is associated with psychological strains for the parents. Parents describe that having a child with CHD leads to a burden of uncertainty and is overall a life-changing experience [5].

Consequently, parents of children with CHD have a high prevalence of posttraumatic stress disorder symptoms (30%), depression and anxiety (25–50%), and severe psychological distress (30–80%) [6]. Further, a systematic review demonstrated diminished health-related QoL (HRQoL) and general QoL in parents of children with CHD compared to parents of healthy children and children with other illnesses. QoL is a multidimensional construct for the subjective perception of affect and satisfaction with life across physical, social, and psychological domains of daily life [7]. Factors that contribute to low QoL include stress, anxiety, depression, anger, hopelessness, sense of isolation, inadequate social support sources, financial burden, decreased vitality, impaired physical functioning, and sleeping problems. These factors most likely interact with each other and substantially impact the family’s functioning and everyday life. Risk factors previously identified for low QoL include the severity of CHD, lower child’s age, less social support, and fewer financial resources [8]. Also, neurodevelopmental impairments, which are common in children with CHD [9], may add additional burden to their parents’ daily life an may thus impact their QoL.

Most studies investigating well-being (i.e., mental health and HRQoL) in parents of children with CHD have used cross-sectional study designs and investigated well-being during or shortly after hospitalization. Few studies have investigated longitudinal changes in parental well-being. Although these studies have consistently found lower parental well-being during hospitalization and at discharge [10–13], long-term outcomes have been mixed. Two studies showed an improvement of well-being to a normal level six [10] and 12 months [11] after hospital discharge. In contrast to these findings, a nationwide population-based cohort study in Norway found lower subjective well-being (measured as cognitive aspect, positive affect and negative affect) and higher symptoms of depression and anxiety, in mothers of children with complex CHD than in mothers of healthy children at 6, 18, and 36 months after the child’s birth [14, 15]. To date, no study has investigated well-being in parents of children with CHD through the children’s development into adolescence. The complexity of the CHD, the parents’ social situation and potential neurodevelopmental impairments of the children may influence the long-term trajectory of parental well-being.

In this study, we investigated trajectories of psychological dimensions of HRQoL (i.e., mental HRQoL [m-HRQoL]) in mothers of children with CHD from hospital discharge after the first open-heart surgery until the children were 13 years of age. We further aimed to identify classes of maternal m-HRQoL trajectories and risk factors for low m-HRQoL over time.

We hypothesized that we would identify subgroups of trajectories of maternal m-HRQoL including a subgroup of mothers with good m-HRQoL and a subgroup of mothers with persistent poor m-HRQoL. We further wanted to explore whether additional subgroups of mothers experienced worsening or improving m-HRQoL over time. In addition, we hypothesized that having persistent poor m-HRQoL was associated with having a child with a complex CHD (i.e., univentricular CHD, > 1 cardiopulmonary bypass [CPB] surgery), low psychosocial resources (i.e., migration background, low education, sparse social support) and having a child with neurodevelopmental impairments (i.e., low IQ).

## Methods

### Study design and sample

This prospective longitudinal cohort study was conducted at the University Children’s Hospital Zurich, Switzerland. Children with CHD who underwent CPB surgery between 2004 and 2009 and were assessed at six measurement timepoints: at discharge after the first CPB surgery and at the child’s age of 1, 4, 6, 10 years and in adolescence between 11 and 15 years of age (mean age = 13.7 years). At each measurement timepoint, children underwent a neurodevelopmental assessment and parents completed questionnaires on their children’s behavior and HRQoL, family functioning, and their own mental health and HRQoL (see [16, 17] for details).

**Fig. 1:**
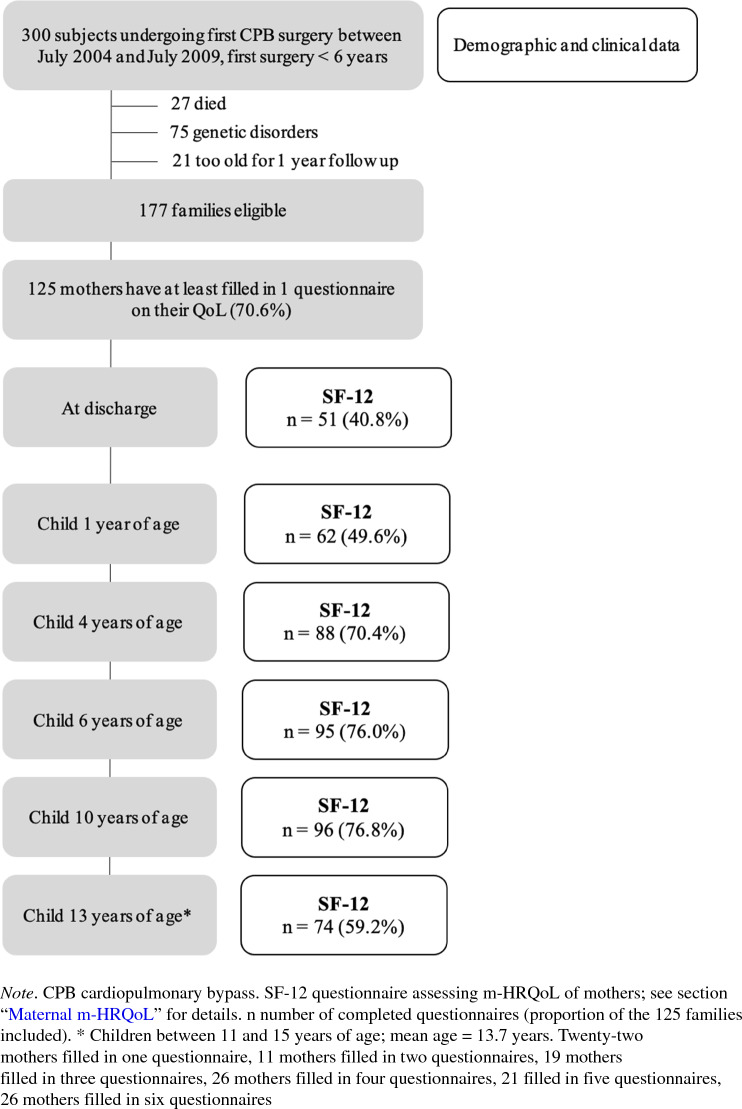
Flowchart of recruitment procedure

For the current analysis, families were included if their child underwent CPB surgery before the one-year assessment (conducted when the child was between 1 year 0 months and 1 year 11 months of age). Further, families were included if their child had no genetic or chromosomal disorder because these children were not assessed after 6 years of age. Therefore, of the 300 families initially included in the prospective cohort study, 177 families were eligible for the current analysis. Of these 177 families, 125 mothers completed a questionnaire about their own m-HRQoL at one or more measurement timepoints and were included in this analysis. The detailed recruitment procedure is displayed in Fig. 1.

### Measures

#### Maternal m-HRQoL

Maternal m-HRQoL was assessed with the mental subscale of the 12-item Short Form-12 Health Survey (SF-12) [18]. The SF-12 is a highly reliable short version of the Medical Outcomes Study Short Form-36 item questionnaire (SF-36) (*r* = 0.57–0.94). The mental subscale of the SF-12 assesses four dimensions: vitality, social function, role limitations due to emotional problems, and mental health. A mental summary score was built in line with that by Wirtz et al. [19].

Standardized z-scores were computed with a normative sample from Switzerland (*n* = 1209 [20]). The raw data on this normative sample was provided upon request by Roser et al. and included the following variables: SF-12 mental component summary score, age, sex, education, migration background (immigrant or non-immigrant), and having children (yes or no). Participants of the normative sample were weighted according to the distribution of sex, age, and nationality to match the distribution of the general population of Switzerland [20]. These weightings were also considered for the current analyses. To match the normative sample to the study sample appropriately, a subset of the normative sample was extracted including only mothers (sex = female, having children = yes) between 19 and 58 years of age (*n* = 264).

#### Predictors for maternal m-HRQoL

Cardiac and clinical data were prospectively collected from patients’ charts. Migration background was assessed at the child’s first hospitalization. Parental education was calculated by maternal and paternal education on a 6-point scale (1 = no high-school degree, 2 = high-school degree, 3 = apprenticeship, 4 = higher diploma for craftsmen or craftswomen, 5 = advanced diploma of higher education, 6 = university degree), ranging from 2 to 12 [21]. Higher scores indicate better parental education, which was used as a proxy for socioeconomic status (SES). Child IQ was assessed at 10 years of age with the German Version of the Wechsler Intelligence Scale for Children, 4th edition [22] and was used as a proxy for the child’s neurodevelopmental functioning. Perceived social support was repeatedly assessed at each measurement timepoint with the self-reported 14-item Social Support Questionnaire (F-SozU-K14) [23]. This questionnaire measures the perceived extent of support from the social network that is accessible if needed and includes three dimensions: emotional support, practical support, and social integration. A sum score was calculated according to the manual, with higher scores indicating more social support.

### Statistical analyses

Analyses were conducted with two statistical software packages, MPlus (Version 8.4; Muthén & Muthén, 2017) and R [24]. Missing data were estimated with imputation by chained equation [25]. Single imputation (m = 1) with 5 iterations was conducted on a dataset including the following data: m-HRQoL, social support (F-SozU-K14) and all demographic data presented in Table 1. Results were validated with an independently imputed dataset with a different seed. All *p*-values below 0.05 were considered statistically significant.

**Table 1:** Sample characteristics of mothers and their children with CHD

Sample size, *N*	125	Missing
**Socio-demographic variables of mothers**		
Mothers’ age at child’s hospitalization^a^, *M* (*SD*)	31.46 (4.97)	0
Living in a relationship/marriage^a^, *n* (%)	121 (97%)	0
Number of children at hospitalization^a^, *Mdn* (*IQR*, *Range*)	2 (1–2, 1–5)	0
Parental education^a^, *Mdn* (*IQR*, *Range*)	8 (8–10, 3–12)	5
Swiss nationality,* n* (%)	102 (82%)	0
**Clinical variables of children with CHD**		
**Patient specific**		
Male sex, *n* (%)	73 (58%)	0
Gestational age in weeks, *M* (*SD*)	39.1 (2.1)	1
Birth weight in g, *M* (*SD*)	3220 (636)	0
Cyanotic CHD, *n* (%)	86 (69%)	0
Univentricular CHD, *n* (%)	20 (16%)	0
5-min Apgar score, *Mdn* (*IQR*, *Range*)	9 (8–9, 1–10)	12
IQ at 10 years of age, *M* (*SD*)	96 (14)	1
**Peri-operative at first CPB surgery**		
Age in month at first CPB surgery, *Mdn (IQR, Range)*	0.95 (0.2–4.7, 0.1–21.9)	0
Use of deep hypothermia, *n* (%)	24 (19%)	0
Extracorporeal circulation time in min., *Mdn (IQR*, *Range)*	160 (119–211, 25–405)	0
**Post-operative at first CPB surgery**		
Days at ICU after first CPB surgery, *Mdn (IQR*, *Range)*	8 (5–13, 2–227)	0
Days of intubation, *Mdn (Range)*	4 (2–6, 0.5–110)	0
Length of hospitalization, days *Mdn (IQR*, *Range)*	24 (16–38, 7–229)	0
Total number of CPB surgeries, *Mdn* (*IQR*, *Range*)	1 (1–2, 1–4)	0

*CHD* congenital heart disease, *CPB* cardiopulmonary bypass. *ICU* intensive care unit. Peri- and post-operative variables refer to the children’s first CPB surgery. *N* number, % = proportion, *M* mean, SD Standard deviation, Mdn Median, *IQR* Interquartile range. Missing = Number of missing data

^a^Variables refer to the children’s first CPB surgery

M-HRQoL in mothers of children with CHD was compared at each measurement timepoint to the matched subset of the Swiss normative sample with a linear regression model that controlled for migration background, parental education, and age. The linear regression was additionally weighted with the weight coefficients reported by [20] by applying the *svyglm* function in *R* to match the distribution of sex, age and nationality of the general Swiss population.

Latent class growth analysis (LCGA) was conducted with data from the mothers of children with CHD using maximum likelihood estimation with MPlus to identify subgroups of mothers’ m-HRQoL trajectories over time. Unconditional models with increasing numbers of class solutions up to four classes were compared according to lowest BIC, entropy > 0.8, and significant Lo–Mendell–Rubin and bootstrap likelihood ratio tests (*p*-value < 0.05) [26]. Only class solutions with classes that included at least 20 subjects were considered to maintain power for further analyses. For the LCGA, we standardized the SF-12 mental summary score with the Swiss normative sample. To do this, z-scores were calculated from the means and standard deviations of two age groups of the Swiss normative sample. Age groups were (1) from 19 to 45 years of age (*n* = 108) and (2) from 46 to 58 years of age (*n* = 144). Forming these age groups enables us to account for higher self-reported m-HRQoL in women > 46 years than in younger women in the Swiss normative population (see [20]).

To analyze predictors of low m-HRQoL trajectories, logistic regression analyses were conducted in R. The subgroups identified by LCGA were included as the dependent variable, and the following factors were included as predictors: social support, parental education, migration background, univentricular CHD, and child’s IQ. Because social support was assessed at each measurement timepoint, LCGA was conducted to identify two groups of good or poor social support over time using the same procedure described above (see details in Supplementary Tables 1and 2). Thus, social support was included in the model as a binary variable. Odds ratio (*OR*) and McFadden* R*^*2*^ were reported as effect sizes for predictors and model fit, respectively. A McFadden *R*^*2*^ between 0.2 and 0.4 indicates a good model fit [27].

## Results

### QoL compared to Swiss normative data

A total of 125 mothers of children with CHD were included in these analyses. Sociodemographic characteristics of the mothers and clinical characteristics of their children with CHD are reported in Table 1. CHD types are presented in Supplementary Table 3. Mothers who completed at least one questionnaire about their own m-HRQoL had significantly higher education than mothers who did not complete (*Mann–Whitney–*U test: *W* = 1603, *p* < 0.001). The children’s CHD complexity (i.e., univentricular vs. biventricular CHD) did not differ between mothers who completed questionnaires and those who did not complete (*X*^*2*^(1) = 0.039, *p* = 0.843). The median time difference between the first CPB surgery and the one-year assessment was 10.9 months.

Comparison with the normative sample revealed significantly lower m-HRQoL in mothers of children with CHD at discharge. Some 42% had clinically relevant low m-HRQoL (≥ 1SD below normative mean). Subsequently, m-HRQoL improved. By the time the children were 1 year old, there was no significant difference between m-HRQoL in mothers of children with CHD and the Swiss normative sample. M-HRQoL in mothers of children with CHD was significantly higher than Swiss norms when the children were 4 years and 6 years old. There was no difference by the time the children had reached 10 years and 13 years. For all models, higher parental education was significantly associated with better m-HRQoL at each measurement timepoint, whereas age and migration background were not significantly associated with m-HRQoL. See statistical estimates in Table 2.

**Table 2:** Linear regression models predicting m-HRQoL separately for each measurement timepoint

	Standardized *β* (CI-95)	Unstandardized *B* (*SE*)	*p*-value
**Discharge**			
Group^**+**^	− 0.30 (− 0.44 to -0.15)	− 6.29 (1.38)	** < .0001**
Migration^++^	− 0.08 (− 0.19 to 0.02)	− 1.94 (1.04)	0.063
Parental education	0.20 (0.10 to 0.30)	2.19 (0.51)	** < 0.001**
Mother’s age	0.13 (− 0.02 to 0.27)	0.13 (0.12)	0.050
**Child 1 year old**			
Group^**+**^	0.14 (− 0.02 to 0.29)	2.49 (1.28)	0.052
Migration^**++**^	− 0.09 (− 0.21 to 0.02)	− 1.87 (0.99)	0.060
Parental education	0.12 (0.00 to 0.24)	1.13 (0.49)	**0.020**
Mother’s age	0.09 (-0.08 to 0.25)	0.08 (0.06)	0.213
**Child 4 years old**			
Group^**+**^	0.27 (0.15 to 0.39)	4.65 (1.07)	** < 0.001**
Migration^**++**^	− 0.07 (− 0.19 to 0.05)	− 1.30 (0.92)	0.157
Parental education	0.18 (0.06 to 0.30)	1.58 (0.45)	** < 0.001**
Mother’s age	0.10 (− 0.06 to 0.25)	0.09 (0.06)	0.110
**Child 6 years old**			
Group^**+**^	0.20 (0.09 to 0.32)	3.59 (1.04)	** < 0.001**
Migration^**++**^	− 0.07 (− 0.19 to 0.04)	− 1.37 (0.94)	0.147
Parental education	0.18 (0.06 to 0.30)	1.62 (0.46)	** < 0.001**
Mother’s age	0.10 (− 0.04 to 0.25)	0.11 (0.06)	0.077
**Child 10 years old**			
Group^**+**^	0.05 (− 0.06 to 0.16)	0.95 (1.03)	0.356
Migration^**++**^	− 0.07 (− 0.18 to 0.05)	− 1.33 (1.01)	0.187
Parental education	0.16 (0.04 to 0.27)	1.49 (0.49)	**0.003**
Mother’s age	0.07 (− 0.06 to 0.19)	0.08 (0.06)	0.212
**Child 13 years old**			
Group^**+**^	0.06 (− 0.04 to 0.16)	1.03 (0.97)	0.287
Migration^**++**^	− 0.09 (− 0.20 to 0.03)	− 1.71 (0.98)	0.081
Parental education	0.17 (0.06 to 0.29)	1.61 (0.48)	** < 0.001**
Mother’s age	0.07 (− 0.05 to 0.20)	0.09 (0.06)	0.163

Groups: mothers of children with CHD (*n* = 125), mothers of the Swiss normative sample (*n* = 264). Significant effects (*p* < 0.05) are displayed in bold

^ +^Reference: mothers of the Swiss normative sample

^++^Reference: no migration background

### LCGA to identify m-HRQoL trajectories

The fit indices for the class solutions of the LCGA are reported in Supplementary Table 4. LCGA confirmed the best model fit for the two-class solution separating m-HRQoL scores of mothers of children with CHD across time. This two-class solution was supported by significant likelihood ratio tests and moderate to good entropy. BIC was comparable to the three-class solution, and class sizes were adequate. The three-class solution was rejected due to an insignificant Lo-Mendell-Rubin likelihood ratio test and too small class sizes. For the four-class solution, the best likelihood value could not be replicated.

**Fig. 2:**
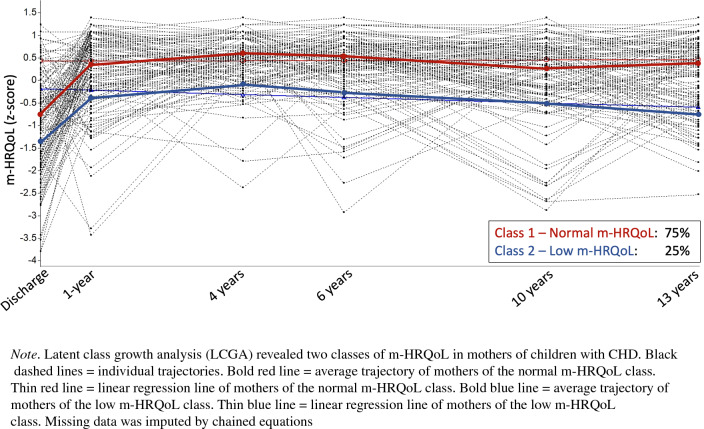
Trajectories of m-HRQoL in mothers of children with CHD

The patterns observed in the two-class solution are meaningful and consistent with a priori hypotheses. The first class was labelled “normal m-HRQoL” (*n* = 94, 75%) and is characterized by average m-HRQoL (mean z-score between −0.76 and 0.62) with no significant linear change over time (slope: *β* (SE) = 0.00 (0.01), *p* = 0.722). The second class was labelled “low m-HRQoL” (*n* = 31, 25%) and is characterized by m-HRQoL below average (mean z-score between −1.32 and −0.10) with a significant linear decrease over time with a small effect size (slope: *β* (SE) = −0.03 (0.02) per year, *p* = 0.040). See means and standard deviations for each class (class 1 = Normal m-HRQoL, class 2 = Low m-HRQoL) and measurement timepoint in Table 3. Average trajectories per class and individual trajectories are displayed in Fig. 2.

**Table 3:** Statistical estimates for each maternal m-HRQoL class and follow-up

	Mean(SD) class 1	Mean (SD) class 2
At discharge	−0.76 (1.05)	−1.32 (1.18)
1-year follow-up	0.41 (0.77)	−0.39 (0.98)
4-year follow-up	0.62 (0.40)	−0.10 (0.75)
6-year follow-up	0.55 (0.51)	−0.32 (0.90)
10-year follow-up	0.29 ()	−0.53 (1.10)
13-year follow-up	0.43 (0.78)	−0.86 (0.71)

Values are based on z-scores. Class 1 = normal m-HRQoL, Class 2 = low m-HRQoL

### Predictors of low m-HRQoL

Logistic regression was conducted to investigate predictors for classes. The low m-HRQoL class was significantly associated with receiving low social support over time. There was no significant association between class assignment and parental education, migration background, number of CPB surgeries, having a child with an univentricular CHD, or low IQ – also when social support was excluded from the model (data not presented). Model fit was low (McFadden *R*^*2*^ = 0.095). Statistical estimates are presented in Table 4.

**Table 4:** Multivariable logistic regression model predicting assignment to “low m-HRQoL class”

	*OR* (*CI*-95)	*p*-value
Social support	0.29 (0.12–0.71)	**0.008**
Parental education	0.92 (0.71–1.18)	0.533
Migration background^+^	1.83 (0.63–5.19)	0.257
Number of CPB surgeries	1.28 (0.67–2.35)	0.432
Child with univentricular CHD^++^	2.59 (0.54–15.654)	0.258
Child IQ	1.02 (0.98 to 1.07)	0.260

^+^Reference: no migration background

^++^Reference: univentricular CHD

## Discussion

This is the first study to longitudinally investigate psychological dimensions of HRQoL (m-HRQoL) in mothers of children with CHD over a period of 13 years. We found that m-HRQoL in mothers of children with CHD after their children’s first open-heart surgery was significantly lower than in a matched normative group of mothers. At discharge, 42% of mothers reported clinically relevant impairment of m-HRQoL. Subsequently, maternal m-HRQoL improved to a normal range (at child’s age 1, 10, and 13 years) and even above normal range (child’s age at 4, and 6 years). However, a large variability in m-HRQoL outcomes was observed, and LCGA identified two groups from differing trajectories: a subgroup with normal m-HRQoL (75% of mothers) and a subgroup with low m-HRQoL over time (25% of mothers). Sparse social support was associated with a more than three-fold increased risk of low m-HRQoL in mothers of children with CHD.

### M-HRQoL of mothers of children with CHD compared to normative data

We found mothers of children with CHD to be at risk for low m-HRQoL when their child undergoes open-heart surgery. This is in line with previous studies [10–13]. During the period of hospitalization and surgery, parents face several psychological strains, including the fear of losing their children, bonding issues, simultaneous care for other family members, and financial burdens [3, 4]. Beside low m-HRQoL, previous studies also reported symptoms of posttraumatic stress, anxiety, and depression during the child’s hospitalization and shortly after [6]. Another study showed that 16% of mothers and 13% of fathers assessed with a self-rated screening questionnaire met diagnostic criteria for a posttraumatic stress disorder after the child’s first open-heart surgery [28].

In our study, low-maternal m-HRQoL at discharge improved to a normal or even above-normal range at the child's age of 1 to 13 years. However, although an overall improvement in maternal m-HRQoL occurred, m-HRQoL varied substantially over time. Studies with short-term follow-ups have reported mixed findings. Longitudinal studies found that initially low parental HRQoL improved to normal levels 6 months after discharge, and increased anxiety during hospitalization eventually improved to normal levels > 12 months after discharge [10, 11]. In contrast, other studies have found persistently lower well-being and increased depressive and anxiety symptoms during early childhood [14, 15, 29]. A cross-sectional study including parents of children with CHD attending preschool showed substantial variation in parental psychological distress, with 27% of mothers reporting high levels of stress and 19% reporting defensive low levels of stress [30].

Improvement of parental well-being after a traumatic and stressful event, such as having a hospitalized child undergoing open-heart surgery, could result from a range of psychological processes. First, parents could develop and adopt coping strategies to adjust to the cardiac condition of their child and associated stressors. Indeed, Demianczyk et al. used semi-structured interviews to identify frequently used coping strategies in parents of children with CHD. Coping strategies include amongst others (1) self-care to help reduce stress, termed active coping, (2) seeking emotional support among their social network, (3) accepting the situation and trying to find peace, and (4) being spiritual and having faith. In Demianczyk et al.’s study, parents also reported coping strategies that are unique to having a critically ill child, such as self-education, trust in the medical team, connecting with other parents of children with CHD, ensuring presence at the bedside, and spending time away from the bedside for self-care [31]. Another reason for improved m-HRQoL over time could be that parents may develop “posttraumatic growth”: positive personality changes after a life-changing event [32]. Indeed, a study recently demonstrated that parents of children with CHD report moderate to strong posttraumatic growth arising from their experience with CHD [33]. However, associations between posttraumatic growth and improved well-being have not been investigated and therefore warrant further research. Lastly, a “response shift” could be another psychological process explaining improved parental well-being. A response shift indicates that someone has adapted their internal standards and values of well-being after experiencing a traumatic event; the phenomenon has previously been introduced as accompanying improved HRQoL in parents of children with CHD after open heart surgery [10].

Interestingly, in our study, mean m-HRQoL in mothers of children with CHD was even higher than the Swiss norms when the child was in preschool (i.e., 4 and 6 years of age). One explanation for such a “honeymoon phase” could be that in this phase, most children are in good cardiac condition and the burden of potential neurodevelopmental problems may only manifest later at school age, when cognitive and social demands increase. Therefore, parents might feel relieved to return to a normal life and therefore feel exceptionally well during this period in comparison to the stressful time during their children’s hospitalization and open-heart surgery. Again, adaptive coping strategies, posttraumatic growth, and response shift may foster positive well-being during this phase. However, this hypothesis has not been tested, and future qualitative studies may usefully explore age-related changes in the subjective well-being of parents of children with CHD.

### Different trajectories of m-HRQoL over time and risk factors for low m-HRQoL

We found substantial variation in m-HRQoL among mothers of children with CHD. Indeed, LCGA identified two qualitatively distinct subgroups of m-HRQoL trajectories: 75% of mothers belonged to a normal m-HRQoL subgroup, and 25% of mothers belonged to a low m-HRQoL subgroup. In the low m-HRQoL subgroup, m-HRQoL significantly decreased over time with a relatively small effect size. This finding underlines the need to identify those at risk for low and decreasing m-HRQoL to provide early psychosocial support. We found that persistent low m-HRQoL was associated with sparse social support. This is in line with previous studies that identified social support as one of the most consistent and strongest predictors of parental well-being and resilience [34]. Another study found that a combination of family-related predictors, which include dyadic adjustment, social support, parenting stress, and posttraumatic stress symptoms, explained approximately 75% of the variance in QoL outcome 4 months after discharge [35]. A systematic review summarized how the family factors of family strain, parental perceptions, and coping were stronger predictors of parental well-being than the illness severity and physical limitations of the child [8]. In agreement with their finding, we did not observe an association between low m-HRQoL over time and CHD severity measured as number of surgeries and univentricular CHD. Lotto and colleagues suggest that the parental perception of CHD severity differs from the CHD severity defined by the cardiologists. They found poor agreement between parental risk estimation and objective measures of surgical risk [36]. Indeed, a parent’s subjective appraisal of their child’s health may affect their well-being more strongly than the actual clinical risk. Future studies should consider this difference when evaluating predictors of parental well-being.

25% of mothers of children with CHD reported chronically low m-HRQoL. Further studies are needed to evaluate how these mothers can be supported and investigate the impact of family-based interventions on their long-term well-being. The importance of parental well-being has been underlined by research showing that low maternal mental health is associated with neurodevelopmental impairments in children with CHD [37–39]. Few studies have investigated interventional approaches to supporting mental health in parents of children with CHD. A systematic review demonstrated that parent interventions during the children’s stay on the ICU may improve maternal coping, mother–infant attachment, parenting confidence, satisfaction with clinical care, and infant mental health (see systematic review by [40]). Another study demonstrated that skin-to-skin care during the stay on the ICU can mitigate the negative effects of parental role alteration by reducing maternal physiological and psychological stress responses. This was observed in reduced salivary cortisol levels and anxiety symptoms [41]. Two randomized controlled trials showed that educational intervention programs after hospitalization improved maternal QoL and anxiety symptoms [42, 43]. These studies demonstrate promising interventional approaches to promoting parental mental health during and after hospitalization. Nevertheless, health policies that prioritize parental mental health care for parents with critically ill children remain lacking [40]. Further, reasons why parents do not proactively seek mental health support can be manifold and include (a) the belief that psychological support is outside the care team’s scope of practice, (b) the inclination to “stay strong” and fear negative judgment, and (c) the desire to focus care resources on the child [44]. Thus, substantial effort and standardized screening procedures may be required by the clinical care team to facilitate mental health support for parents of children with CHD.

Our study has shown that many mothers report critically poor m-HRQoL at the time of hospitalization. Although some of these mothers recover and report normal m-HRQoL throughout their child’s development, others continue to have low m-HRQoL. We have shown that CHD complexity may not adequately predict the trajectory of maternal m-HRQoL, but perceived social support can. Screening for psychosocial resources during hospitalization may be suitable for early identification of mothers at risk for persistent low m-HRQoL. Future studies are needed to investigate screening tools for identifying mothers at risk to target psychological interventions on those who need them most.

## Limitations

This longitudinal study used m-HRQoL data on mothers of children with CHD, compared their m-HRQoL to matched normative data, and investigated the prediction of two m-HRQoL trajectories. However, some limitations should be taken into account when interpreting our results. First, mothers included in this analysis had significantly higher education than mothers who did not complete any m-HRQoL questionnaires. Therefore, our results may not be fully generalizable to the whole population of mothers of children with CHD. Second, we used m-HRQoL to measure maternal psychological adjustment over time. Thus, our results cannot provide any information about specific mental health problems, such as depression, anxiety, and posttraumatic stress. Future longitudinal studies are needed to investigate specific psychological disorders and thus select specific treatments and support. Further, we have not collected data on maternal m-HRQoL during pregnancy, but approximately 40% of CHD diagnoses are made prenatally [45]. Parents with a prenatal CHD diagnosis may be more deeply affected than parents who receive a postnatal CHD diagnosis [46]. The long-term effects of impaired mental health during pregnancy warrant further investigation and are important for defining the optimal timing of mental health interventions for parents of children with CHD.

Another limitation is that the normative data used for comparison with mothers of children with CHD was cross-sectional. Thus, we were unable to compare longitudinal trajectories between mothers of children with CHD and mothers of healthy children. Nevertheless, we were able to use a matched normative sample, and we corrected for age to control for normal age-related changes in m-HRQoL.

Over the course of 13 years, missing m-HRQoL data was relatively high. 58% of mothers filled in 4 or more questionnaires. Missing data may introduce bias. To maintain power and account for relations in the data, missing data was estimated with imputation by chained equation [25].

Lastly, we only investigated mothers of children with CHD and did not include fathers in this analysis because we lacked sufficient responders. Previous cross-sectional studies have found that fathers of children with CHD also report low m-HRQoL and symptoms of posttraumatic stress, depression, and anxiety [6, 8]. Nevertheless, fathers are substantially underrepresented in studies investigating parental well-being [8]. In addition, we did not assess siblings and we did not assess if siblings had any chronic conditions. Future studies should also include siblings of children with CHD, because their well-being may also be substantially affected and they could further influence parental outcome.

## Conclusion

In mothers of children with CHD, trajectories of m-HRQoL from hospital discharge throughout the child’s development are heterogenous. A subgroup of mothers with continuously low m-HRQoL was identified. Overall, mothers of children with CHD are at risk for low m-HRQoL in the initial period when their children undergo open-heart surgery. Importantly, a significant subgroup of mothers continues to have low m-HRQoL throughout their children’s development into adolescence. Sparse social support was associated with an increased risk of low m-HRQoL over time, whereas CHD severity was not predictive of m-HRQoL. Studies of family-oriented approaches targeting families with low psychosocial support are needed to reinforce parental well-being in the long term. Future longitudinal studies investigating parental well-being should also consider fathers and siblings, because they are often underrepresented in mental health studies and may have different HRQoL trajectories from mothers with, consequently, differing needs.

## Practical implications

The current study findings provide evidence that a subgroup of mothers of children with CHD continues to be at long-term risk for low m-HRQoL. These findings indicate that screening of parental HRQoL and mental health problems remain important beyond the time of CPB surgery. Implementing screenings for parental mental health during cardiac or neurodevelopmental follow-up consultations could help identifying and supporting families in need. Further research on and subsequent implementation of family-based interventions are needed in the care of children with CHD.

## Supplementary Information

Below is the link to the electronic supplementary material.

Supplementary file1 (DOCX 17 kb)

## Data Availability

De-identified data are available from the corresponding author upon reasonable request.

## Abbreviations

CHD: Congenital heart disease
QoL: Quality of life
ICU: Intensive care unit
SF-12: Short Form-12 Health Survey
F-SozU-K14: 14-Item Social Support Questionnaire
LCGA: Latent class growth analysis
