# Supplementary material for: Mental health-related quality of life in mothers of children with surgically repaired congenital heart disease: a 13-year longitudinal study
Source: Qual Life Res. 2023 May 30;32(10):2975–86. doi: 10.1007/s11136-023-03440-y (PMC10474212; doi:10.1007/s11136-023-03440-y)
Supplement: Supplementary file 1 — Supplementary file1 (DOCX 17 kb) [file 11136_2023_3440_MOESM1_ESM.docx]

**Supplementary Materials**

**Supplementary Table 1:** Model fit comparison for LCGA of perceived social support

| Number of classes per model | Bayesian information criteria | Lo-Mendell-Rubin likelihood ratio test (*p*-value) | Bootstrapped likelihood ratio test  (*p*-value) | Entropy | Average latent class probabilities | Proportions for the latent classes |
| --- | --- | --- | --- | --- | --- | --- |
| **2** | **5894.6** | **<0.001** | **<.001** | **0.850** | **0.924-.978** | **0.38-0.62** |
| 3 | 5900.7 | 0.550 | .095 | 0.733 | 0.728-.935 | 0.22-0.56 |
| 4 | 5903.2 | 0.273 | <.001 | 0.748 | 0.724-.920 | 0.08-0.48 |

*Notes.* Values are based on the FSozU assessed at the children’s ages of 1, 4, 6, 10, and 13 years.

**Supplementary Table 2:** Latent class growth model estimates for perceived social support

|  | n | unstandardized *B* | standard error | *p*-value |
| --- | --- | --- | --- | --- |
| **Class 1** | 47 |  |  |  |
| Intercept |  | 32.994 | 6.788 | <.001 |
| Slope |  | 1.073 | 0.784 | 0.171 |
| **Class 2** | 78 |  |  |  |
| Intercept |  | 74.675 | 3.744 | <.001 |
| Slope |  | 0.310 | 0.501 | 0.536 |

*Notes.* *n* = numbers of mothers in the respective latent class.

**Supplementary Table 3:** Types and frequencies of CHD diagnoses

| Diagnoses | *N (%)* |
| --- | --- |
| **Biventricular heart defects** |  |
| Transposition of the great arteries (TGA) | 36 (29%) |
| Ventricular septal defect (VSD) | 19 (15%) |
| Tetralogy of Fallot (TOF) | 15 (12%) |
| Truncus arteriosus (TA) | 5 (4%) |
| Coarctation | 7 (6%) |
| Atrioventricular canal defect (AVCD) | 6 (5%) |
| Total anomalous pulmonary venous connection (TAPVC) | 4 (3%) |
| other | 13 (10%) |
|  | 105 (84%) |
| **Univentricular heart defects** |  |
| Pulmonary atresia (PA) | 2 (2%) |
| Hypoplastic left heart syndrome (HLHS) | 8 (6%) |
| Other functional single ventricle anomaly | 10 (8%) |
|  | 20 (16%) |

**Supplementary Table 4**: Model fit comparison for the LCGA of m-HRQoL

| Number of classes per model | Bayesian information criteria | Lo-Mendell-Rubin likelihood ratio test (*p*-value) | Bootstrapped likelihood ratio test  (*p*-value) | Entropy | Average latent class probabilities | Proportions for the latent classes |
| --- | --- | --- | --- | --- | --- | --- |
| **2** | **1951.1** | **0.006** | **<0.001** | **0.744** | **0.884-0.949** | **0.25-0.75** |
| 3 | 1950.2 | 0.266 | <0.001 | 0.794 | 0.897-0.924 | 0.02-0.72 |

*Notes.* Values are based on the SF-12 assessed at discharge, and the children’s ages of 1, 4, 6, 10, and 13 years. The best likelihood value for the four-class solution could not be replicated.
